# Knockout of the entire family of *AITR* genes in Arabidopsis leads to enhanced drought and salinity tolerance without fitness costs

**DOI:** 10.1186/s12870-021-02907-9

**Published:** 2021-03-16

**Authors:** Siyu Chen, Na Zhang, Ganghua Zhou, Saddam Hussain, Sajjad Ahmed, Hainan Tian, Shucai Wang

**Affiliations:** 1 Laboratory of Plant Molecular Genetics & Crop Gene Editing, School of Life Sciences, Linyi University, 276000 Linyi, China; 2 Key Laboratory of Molecular Epigenetics of MOE, Northeast Normal University, 130024 Changchun, China

**Keywords:** ABA, Abiotic stress, AITRs, Arabidopsis, Gene editing, Fitness cost

## Abstract

**Backgorund:**

Environmental stresses including abiotic stresses and biotic stresses limit yield of plants. Stress-tolerant breeding is an efficient way to improve plant yield under stress conditions. Genome editing by CRISPR/Cas9 can be used in molecular breeding to improve agronomic traits in crops, but in most cases, with fitness costs. The plant hormone ABA regulates plant responses to abiotic stresses via signaling transduction. We previously identified AITRs as a family of novel transcription factors that play a role in regulating plant responses to ABA and abiotic stresses. We found that abiotic stress tolerance was increased in the single, double and triple *aitr* mutants. However, it is unclear if the increased abiotic stress tolerance in the mutants may have fitness costs.

**Results:**

We report here the characterization of *AITRs* as suitable candidate genes for CRISPR/Cas9 editing to improve plant stress tolerance. By using CRISPR/Cas9 to target *AITR3* and *AITR4* simultaneously in the *aitr256* triple and *aitr1256* quadruple mutants respectively, we generated Cas9-free *aitr23456* quintuple and *aitr123456* sextuple mutants. We found that reduced sensitivities to ABA and enhanced tolerance to drought and salt were observed in these mutants. Most importantly, plant growth and development was not affected even in the *aitr123456* sextuple mutants, in whom the entire *AITR* family genes have been knocked out, and the *aitr123456* sextuple mutants also showed a wild type response to the pathogen infection.

**Conclusions:**

Our results suggest that knockout of the *AITR* family genes in Arabidopsis enhanced abiotic stress tolerance without fitness costs. Considering that knock-out a few *AITRs* will lead to enhanced abiotic stress tolerance, that AITRs are widely distributed in angiosperms with multiple encoding genes, *AITRs* may be targeted for molecular breeding to improve abiotic stress tolerance in plants including crops.

## Background

The world population is expected to reach 9 billions by the year 2050, as a consequence, a 70 % increase in crop yield are needed in order to feed the population by then [1, 2]. However, yield of plants including crops is largely affected by environmental stresses, including abiotic stresses such as drought, salinity and low and/or high temperatures, and biotic stresses such as pathogens and insects [3, 4]. It is estimated that biotic stresses cause an average ~ 20 % global yield loss for most major crops, whereas abiotic stresses, ~ 50 % [5, 6]. Among the abiotic stresses, drought and salinity are occurred in many different regions, and it is predicted that more than 50 % of all arable lands on the earth will be salinized seriously by the year 2050 [3]. Accelerated climate changes and global warming will aggravate the situation, and lead to a further yield loss for plants including crops.

Breeding for crops with enhanced tolerance to abiotic stresses will increase crop yield under stress conditions, however, traditional breeding takes quite a long time, and the results are usually unpredictable [7]. Molecular breeding, on the other hand is able to largely overcome these shortages [8], whereas new developed techniques, if used properly, may help to further shorten the breeding process.

Shortly after the discovery that the endonuclease Cas9 (CRISPR -associated protein 9) is able to cleave targeted double-stranded DNA in eukaryotic cells [9, 10], CRISPR (clustered regularly interspaced short palindromic repeats)/Cas9 has been developed as a new technique and used for targeted genome editing, and has been used successfully in plants [11–13]. Currently, there are two different types of CRIPSR/Cas9 genome editing systems, i.e., the DNA cleavage system, and the base editor system. The first allows small deletions or insertions of nucleotides at the target sites [14], whereas the second enables generation of precise single-nucleotide substitution [15]. Both of them have been used to generate mutations in plants, not only in the model plant Arabidopsis, but also in some major crops such as wheat and rice, at least in some of the cases, to improve agronomic traits [16–19]. CRISPR/Cas9 genome editing enables to generate predicted mutations, and transgene-free mutants can be isolated from genome edited transgenic plants, therefore can be used to accelerate plant breeding process [17, 19–22]. Recent improvement on the CRISPR/Cas9 systems, such as expanding target space by using Cas9 variants or engineered Cas9 further expanded it capacity for using in plants breeding [23, 24]. However, appreciate candidate genes that can be targeted by CRISPR/Cas9 editing to improve plant abiotic stress tolerance in crop breeding are largely unidentified.

The plant hormone ABA (abscisic acid) is a key stress hormone, it can regulates plant responses to abiotic stresses such as drought, salinity, cold and heat via signaling transduction [25–30]. Several different types of proteins including the receptor proteins PYR1 (Pyrabactin resistance 1)/PYL (PYR1-like)/ RCAR (Regulatory component of ABA receptor), the protein kinases PP2Cs (A-group PROTEIN PHOSPHATASE 2 C) phosphatases, the SnRK2s (NONFERMENTING 1 (SNF1)-RELATED PROTEIN KINASES), and the bZIP (basic region leucine zipper) transcription factors ABF/AREB/ABI5 function as key regulators in ABA signaling [26, 30–35]. Whereas several UBQ ligases can affect protein stability of the key regulators, therefore involve in regulating ABA signaling. As examples, the E2 ligase VPS23A and the E3 ligases CUL4 and RSL1 are able to target the PYR/PYL/RCABR receptors for proteasomal degradation [36–39], the E3 ligases KEG (KEEP ON GOING), DWA1 and DWA2 are able to target ABF/AREB/ABI5 transcription factors for degradation [40–43], and the E3 ligases PUB12 and PUB13 are able to target PP2C protein kinases for degradation [44]. Because ABA plays a key role in regulating plant abiotic stress responses, expression level changes in the ABA signaling regulator genes usually affect plant abiotic stress tolerance. For example, drought tolerance was reduced in the loss-of-function mutants of the ABF/AREB/ABI5 transcription factor genes or the *SnRK2s* genes [45, 46], whereas enhanced drought tolerance was observed in the transgenic plants overexpressing *PYL* genes [47, 48]. Therefore, gene editing of these regulator genes by CRISPR/Cas9 may not able be to improve abiotic tolerance in plants.

In previous report, we identified AITRs (ABA-induced transcription repressors) as an angiosperm conserved novel family of transcription factors, and we found that AITRs function as negative regulators in regulating ABA signaling and plant response to abiotic stresses [30]. We report here the characterization of *AITRs* as proper genome editing targets for improving plant abiotic stress tolerance. We generated Arabidopsis *aitr* mutants with all the six *AITR* genes being knocked out, and found that the mutants showed enhanced drought and salt tolerance, but plant growth and development, and plant response to pathogen infections remaining unaffected in the mutants.

## Results

### Generation of *aitr* mutants with all the *AITR* genes knocked out

We have previously identified AITRs as an angiosperm conserved novel family of transcription factors, and found that tolerance to ABA and abiotic stresses such as drought and salt was enhanced in the *aitr* mutant plants [30], indicating that *AITRs* may be targeted for molecular breeding to increase plant tolerance to abiotic stresses.

In Arabidopsis, there are a total of 6 genes encoding AITRs [30], to further examine the functions of AITRs in plant tolerance to abiotic stresses, and whether loss-of-function of *AITRs* may have fitness costs, we decided to generate high order *aitr* mutants with all the *AITR* genes knocked out. T-DNA insertion mutants were available for *AITR1*, *AITR2*, *AITR5* and *AITR6*, in addition to the *aitr2 aitr5 aitr6* (*aitr256*) triple mutant [30], we thus generated *aitr1 aitr2 aitr5 aitr6* (*aitr1256*) quadruple mutant by crossing. We then generated *aitr2 aitr3 aitr4 aitr5 aitr6* (*aitr23456*) quintuple and *aitr1 aitr2 aitr3 aitr4 aitr5 aitr6* (*aitr123456*) sextuple mutants by using CRISPR/Cas9 to simultaneously target *AITR3* and *AITR4* in the *aitr256* triple and *aitr1256* quadruple mutants, respectively.

**Fig. 1:**
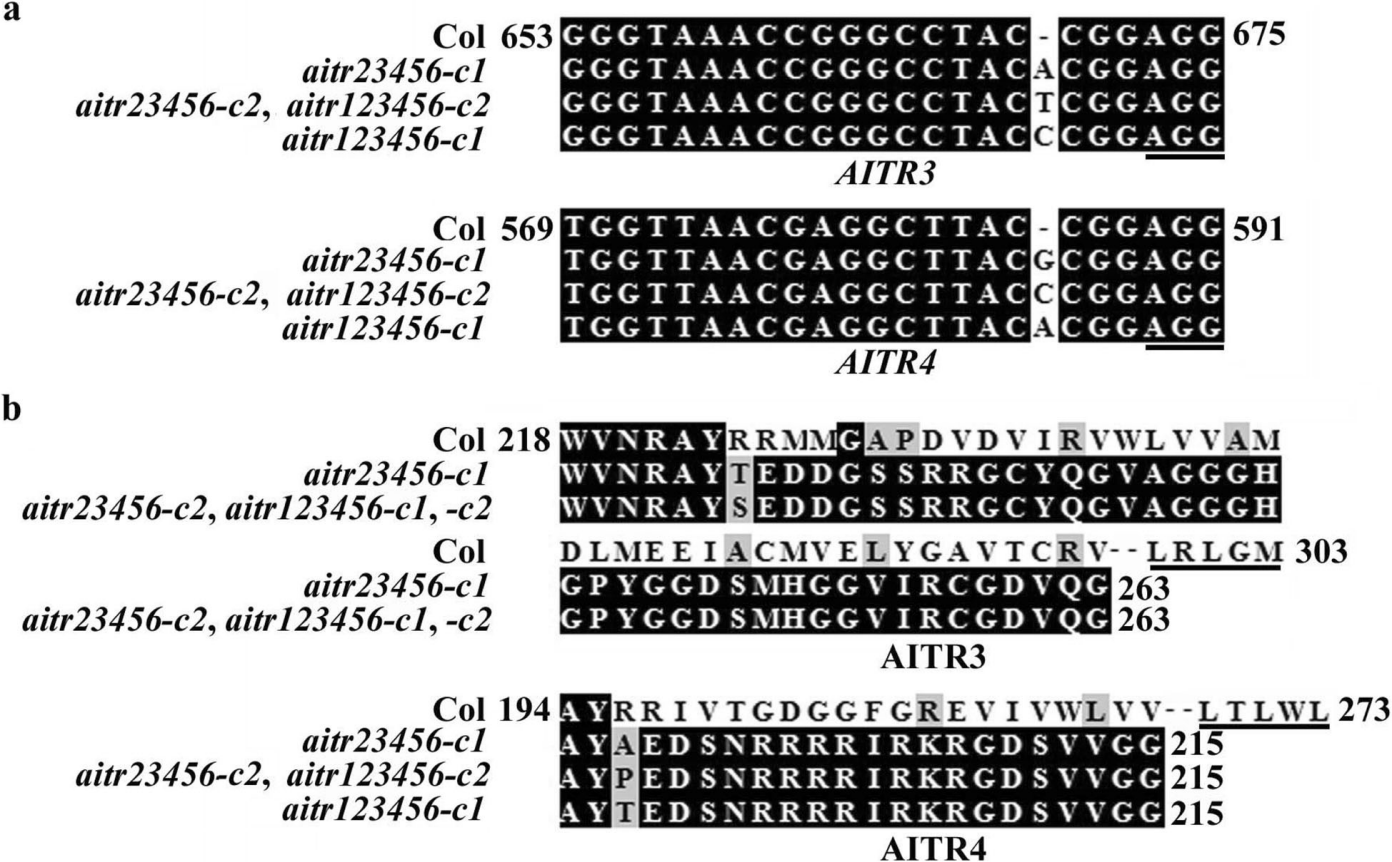
Generation of *aitr23456* quintuple and *aitr123456* sextuple mutants. **a** Alignment of the CRISPR/Cas9 target sequences of *AITR3* and *AITR4* in the Col wild type and the *aitr23456* quintuple and *aitr123456* sextuple mutants. The mutants were generated by transforming the *aitr256* triple and *aitr1256* quintuple T-DNA insertion mutant plants, respectively with a *pHEE* CRISPR/Cas9 construct that simultaneously targeting *AITR3* and *AITR4*. Genome editing status in the selected T1 transgenic plants was examined by sequencing. Homozygous Cas9-free mutants were isolated from T2 or T3 progeny of the individual genome edited T1 plants. Numbers indicate the nucleotide position relative to the start condons of *AITR3* and *AITR4*, respectively. Underlines indicate the PAM sites immediately after the target sites. **b** Amino acid sequence alignment of AITR3 and AITR4 in the Col wild type and the *aitr23456* quintuple and *aitr123456* sextuple mutants. The coding sequences of *AITR3* and *AITR4* in the *aitr23456* quintuple and *aitr123456* sextuple mutants were used for ORF analysis on ORFfinder (https://www.ncbi.nlm.nih.gov/orffinder/). Predicated amino acid sequences were used for alignment with wild type AITR3 and AITR4, respectively. Numbers indicate amino acid position relative to the first Met residue. Underlines indicate the fully or partial conserved LxLxL motif in AITRs

Two impendent Cas9-free homozygous *aitr23456* quintuple mutant lines, i.e., *aitr23456-c1* and *aitr23456-c2*, and two impendent Cas9-free homozygous *aitr123456* sextuple mutant lines, i.e., *aitr123456-c1* and *aitr123456-c2*, were generated. In all the four mutant lines, single nucleotide insertions were occurred at the target sites for both *AITR3* and *AITR4* (Fig. 1a), resulting in frame shift after the nucleotide insertion sites and premature stops of AITR3 and AITR4, respectively (Fig. 1b).

### The *aitr* mutants are hyposensitivity to ABA

In both seed germination and cotyledon greening assays, reduced sensitivity to ABA was observed for all the single T-DNA insertion mutants of genes *AITR1*, *AITR2*, *AITR5* and *AITR6*, and further reduced ABA sensitivity was observed in the double mutants *aitr2 aitr5* (*aitr25*) and *aitr2 aitr6* (*aitr26*), and triple mutant *aitr256* [30], suggesting that AITRs may have redundant functions in regulating ABA responses.

**Fig. 2:**
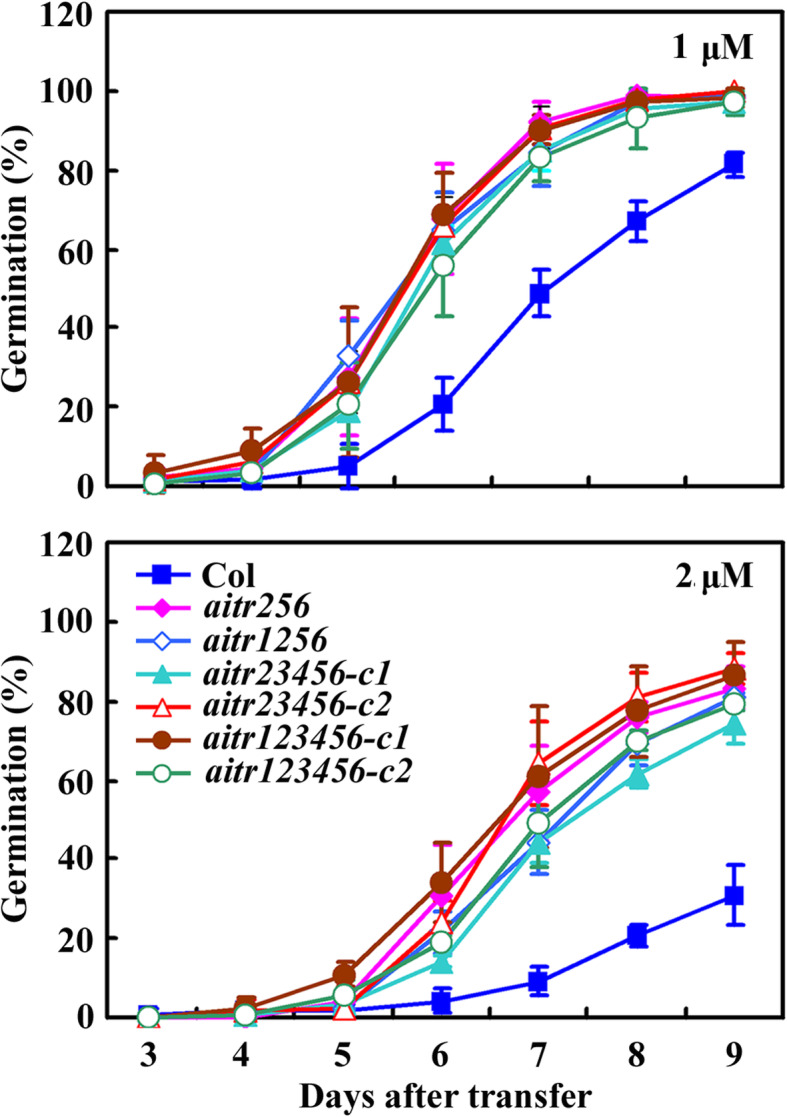
Effects of ABA on seed germination of the *aitr* mutants. Seeds of the Col wild type, the *aitr256* triple, *aitr1256* quadruple, *aitr23456* quintuple and *aitr123456* sextuple mutants were sterilized and sown on 1/2 MS plates in the presence or absence of 1 µM (up panel) or 2 µM ABA (low panel). The plates were transferred, after kept at 4 °C in darkness for 2 days, to a growth room. All the seeds on plates in the absence of ABA were germinated one day after the transfer. Seed germination on ABA-containing plates was examined daily after the transfer, germinated seeds were scored, and percentage of germination was calculated. The experiments were repeated three times, and similar results were obtained. Data represent the mean ± SD of three replicates

Seed germination and cotyledon greening assays were used to examine ABA responses of the *aitr* higher order mutants generated. As shown in Fig. 2, in seed germination assays, all the mutants examined including the *aitr256* triple, *aitr1256* quadruple, *aitr23456* quintuple and *aitr123456* sextuple mutants showed greatly reduced sensitivity to ABA at both the concentrations tested. However, little, if any difference on the germination rate was observed between the *aitr123456* sextuple and the *aitr256* triple mutants (Fig. 2).

**Fig. 3:**
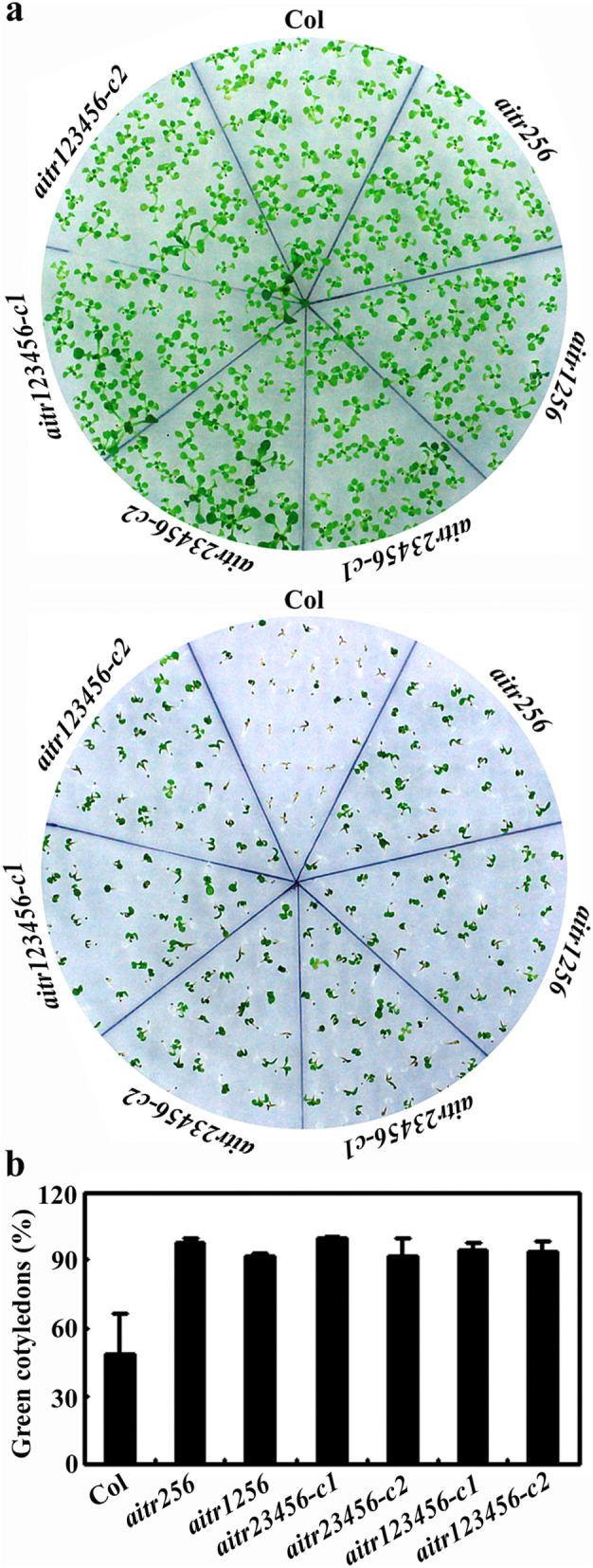
Effects of ABA on cotyledon greening of the *aitr* mutants. **a** Cotyledon greening of the Col wild type and the *aitr* mutants in response to ABA treatment. Seeds of the Col wild type, the *aitr256* triple, *aitr1256* quadruple, *aitr23456* quintuple and *aitr123456* sextuple mutants were sterilized and sown on 1/2 MS plates in the presence or absence of 2.5 µM ABA. The plates were transferred, after kept at 4 °C in darkness for 2 days, to a growth room. Pictures were taken 17 days after the transfer. **b** Quantitative assays of cotyledon greening of the Col wild type and the *aitr* mutants in response to ABA. Green seedlings were scored 17 days after the transfer, and percentage of green seedlings was calculated. The experiments were repeated three times, and similar results were obtained. Data represent the mean ± SD of three replicates

All the mutants examined also showed greatly reduced sensitivity to ABA in cotyledon greening assays (Fig. 3a). Almost all the seedlings of all the mutants produced green cotyledons at the presence of ABA, i.e., nearly 100 % green cotyledons for all the mutant seedlings, compared with ~ 50 % for the Col wild type seedlings (Fig. 3b).

**Fig. 4:**
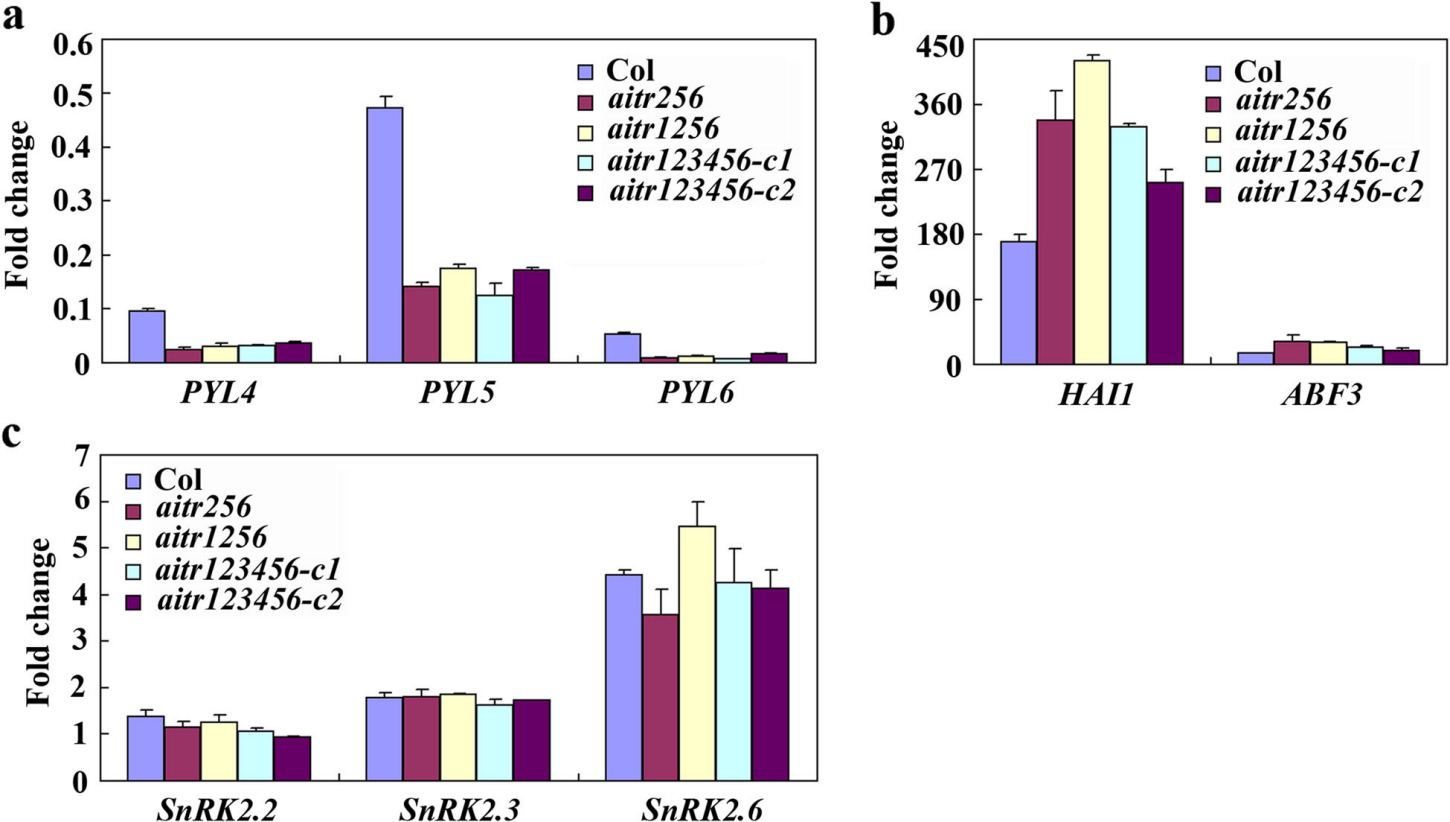
ABA response of some ABA signaling component genes in the Col wild type and the *aitr256*, *aitr1256* and *aitr123456* mutants. **a** Fold changs of the PYR/PYL receptor gene *PYL4*, *PYL5* and *PYL6*. **b** Fold changs of PP2C phosphatase gene *HAI1* and ABF transcription factor gene *ABF3*. **c** Fold changs of SnRK2 kinase gene *SnRK2.2*, *SnRK2.3* and *SnRK2.6*. Twelve-day-old seedlings of the Col wild type and the *aitr256*, *aitr1256* and *aitr123456* mutants were treated with 50 µM ABA, total RNA was isolated and quantitative RT-PCR was used to examine the expression of key regulator genes in ABA signaling. The expression of *ACT2* was used as an inner control, and the fold changes were calculated by comparing the expression levels of the corresponding genes in ABA treated and un-treated samples. The experiments were repeated three times, and similar results were obtained. Data represent the mean ± SD of three replicates

We then compared ABA response of the core ABA signaling regulator genes in the Col wild type, and the *aitr256*, *aitr1256* and *aitr123456* mutants. We found that the expression levels of the PYL receptor genes *PYL4*, *PYL5* and *PYL6* in response to ABA were further decreased in the *aitr* mutants when compare with the Col wild type seedlings, but no difference was observed between the *aitr256*, *aitr1256* and *aitr123456* mutants (Fig. 4a). In contrast, the expression levels of PP2C phosphatase gene *HAI1* and ABF/AREB/ABI5 transcription factor gene *ABF3* in response to ABA were further increased (Fig. 4b), whereas that of the SnRK2 kinase genes *SnRK2.2*, *SnRK2.3* and *SnRK2.6* in response to ABA remained largely unchanged (Fig. 4c).

### The *aitr* mutant plants show enhanced tolerance to drought and salt stresses

**Fig. 5:**
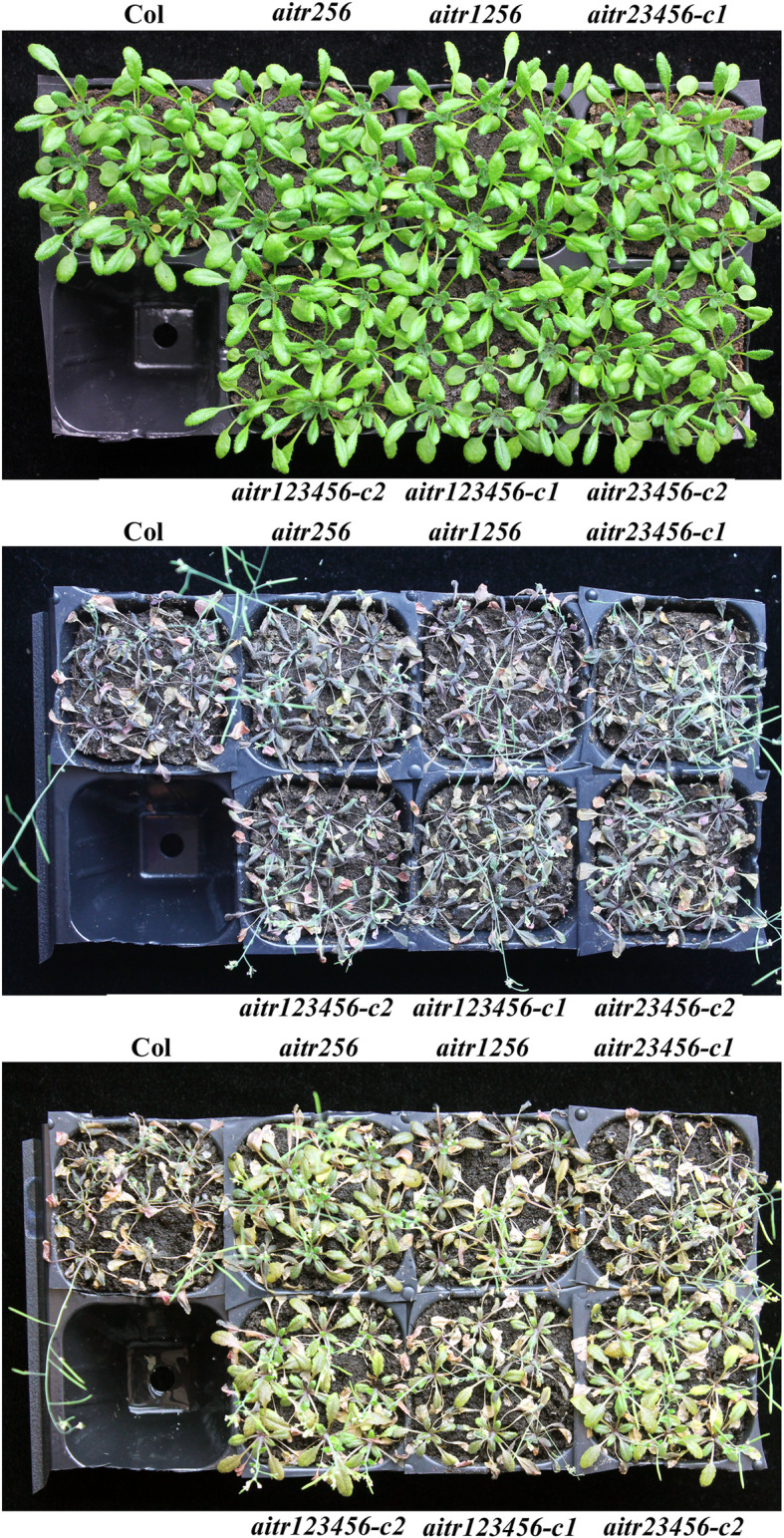
The *aitr* mutants are tolerant to drought treatment. Seeds of the Col wild type, the *aitr256* triple, *aitr1256* quadruple, *aitr23456* quintuple and *aitr123456* sextuple mutants were germinated directly and grown in soil pots in a growth room with sufficient watering for 30 days. The plants were subjected to drought treatment by withholding watering for 12 days. Watering was then resumed. Pictures were taken before (**up panel**), and after drought treatment (**middle panel**), and 2 day after watering was resumed (**low panel**). The experiments were repeated three times, and similar results were obtained.

Expression level changes of the regulator genes in ABA signaling usually affect plant abiotic stress tolerance [45–48]. That is also the case with *AITRs*, the *aitr* single, double and triple mutants examined showed enhanced drought and salt tolerance [30]. We examined abiotic stress tolerance in the high order *aitr* mutants generated. Soil grown mature plants were used for drought and salt treatments. As shown in Fig. 5, after re-watering, most of the mutant plants including the *aitr256* triple, *aitr1256* quadruple, *aitr23456* quintuple and *aitr123456* sextuple mutants were recovered, whereas all the Col wild type plants were not (Fig. 5).

**Fig. 6:**
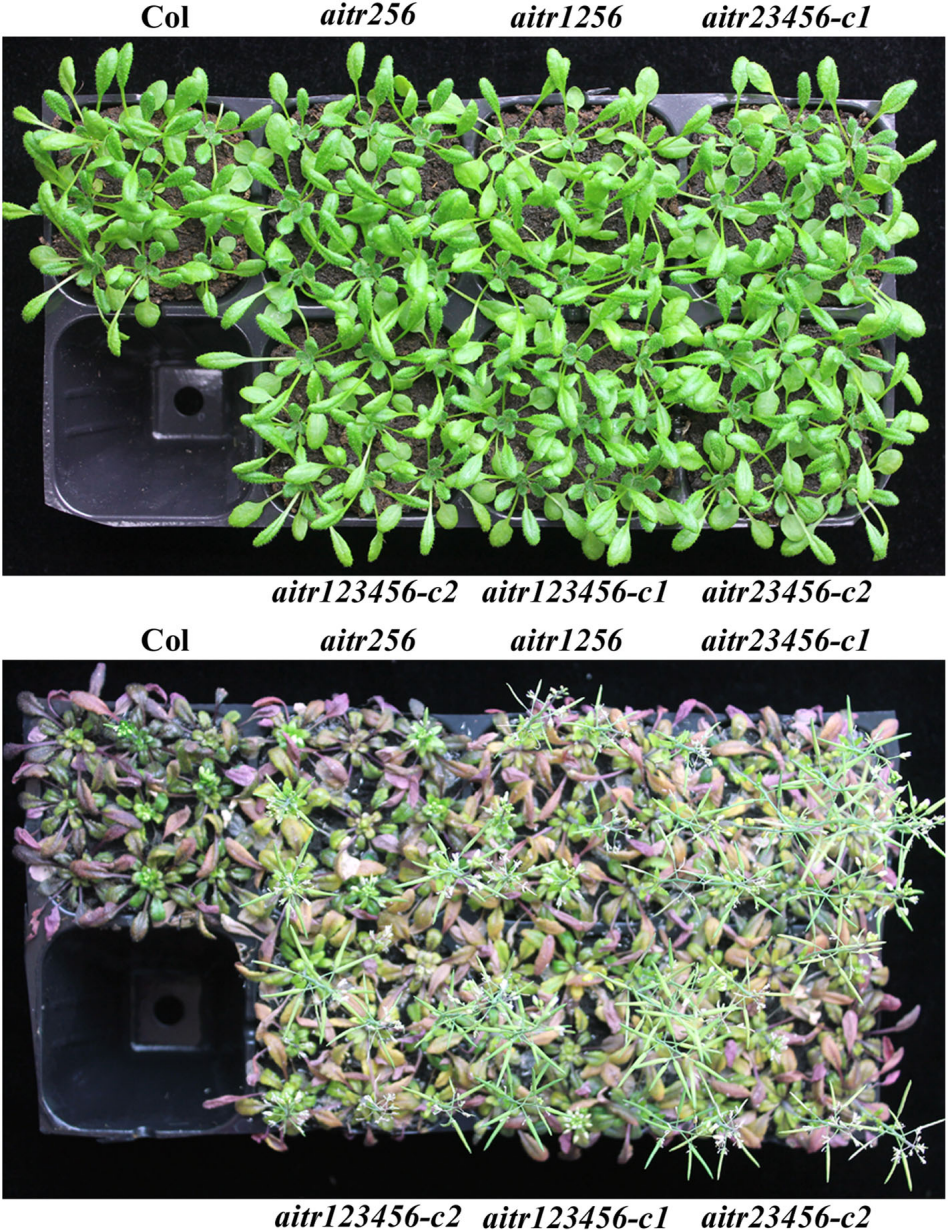
The *aitr* mutants are tolerant to salt treatment. Seeds of the Col wild type, the *aitr256* triple, *aitr1256* quadruple, *aitr23456* quintuple and *aitr123456* sextuple mutants were germinated directly and grown in soil pots in a growth room with sufficient watering for 30 days. The plants were then subjected to salt treatment by watering every other day with 150 mM NaCl for 10 times. Pictures were taken before treatment (**up panel**), and 1 day after the last salt watering (**low panel**). The experiments were repeated three times, and similar results were obtained

All the mutants are also showed enhanced tolerance to salt treatment. As shown in Fig. 6, salt treatment severely affected plant growth and development in the Col wild type plants, which failed to produce seeds. On the other hand, growth and development of the *aitr* mutants including the *aitr256* triple, *aitr1256* quadruple, *aitr23456* quintuple and *aitr123456* sextuple mutants was less affected, as most of the mutant plants were still be able to produce seeds (Fig. 6).

### Growth and development are not affected in the *aitr* mutants

Enhanced stress tolerance usually accompanied by fitness costs on growth and development [49, 50]. The results that enhanced tolerance to abiotic stresses including drought and salt were observed in the *aitr* mutants indicate that *AITRs* may be targeted for plant breeding to enhance plant tolerance to abiotic stresses. However, fitness costs should be evaluated before *AITRs* can be targeted for plant breeding. We therefore examined plant growth and development of the *aitr* mutants under normal growth conditions.

**Fig. 7:**
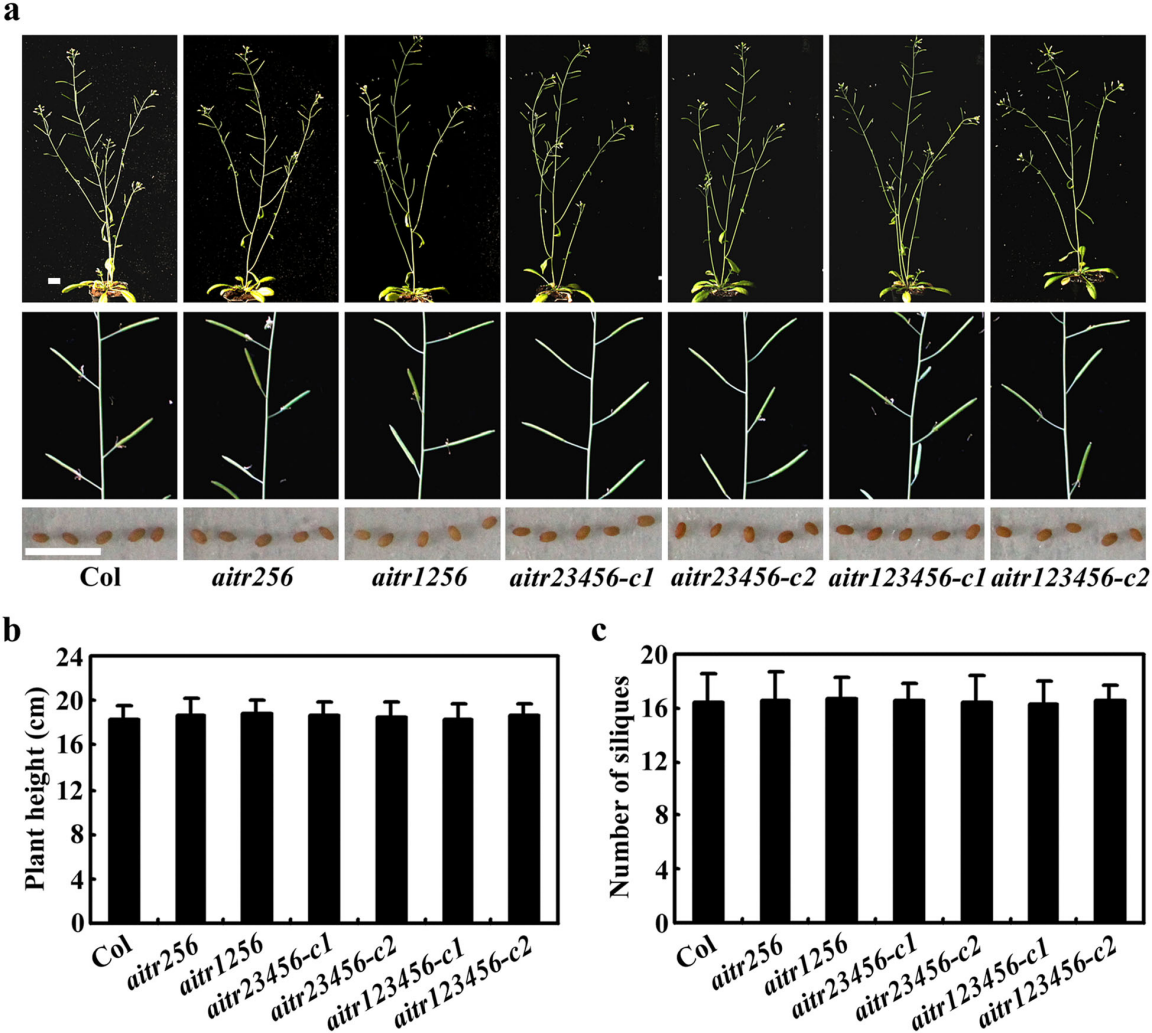
Growth and development of the *aitr* mutants. **a** Plant morphology of the Col wild type and the *aitr* mutants. Up panel, 7-week-old plants, middle panel, main inflorescence stem sliliques, low panel, seeds. **b** Height of the Col wild type plants and the *aitr* mutants. **c** Number of siliques on the main inflorescence stems of the Col wild type and the *aitr* mutants. Seeds of the Col wild type, the *aitr256* triple, *aitr1256* quadruple, *aitr23456* quintuple and *aitr123456* sextuple mutants were generated directly and grown in soil pots in a growth room. Pictures for 7-week-old whole plants and the main inflorescences were taken. Height for 12-week-old plants was measured, and numbers of siliques on the main inflorescence stems were counted. The experiments were repeated three times, and similar results were obtained. Data represent the mean ± SD of 12 plants

For direct comparison, the Col wild type and the *aitr* mutants including the *aitr256* triple, *aitr1256* quadruple, *aitr23456* quintuple and *aitr123456* sextuple mutants were geminated directly and grown in soil pots side by side in a growth room. No obviously different was found between the mutants and the Col wild type plants at all the growth stages during their whole life cycle, including vegetative growth, flowering and seed producing stages (Fig. 7a). Quantitive analysis results show that all the *aitr* mutants reached a same height as that of the Col wild type plants (Fig. 7b), and the numbers of the siliques produced by the plants were also similar (Fig. 7c).

### The *aitr* mutants show a wild type response to pathogen infection

Accumulated experiment evidence suggest that ABA can also plays a role in regulating biotic stress tolerance [51–53], and trade-offs between biotic and abiotic stress responses were observed [54, 55]. Having shown that AITRs are able to regulate ABA response and enhanced tolerance to abiotic stresses including drought and salt were observed in the *aitr* mutants, we examined if plant response to pathogen infection may be affected in the *aitr* mutants.

**Fig. 8:**
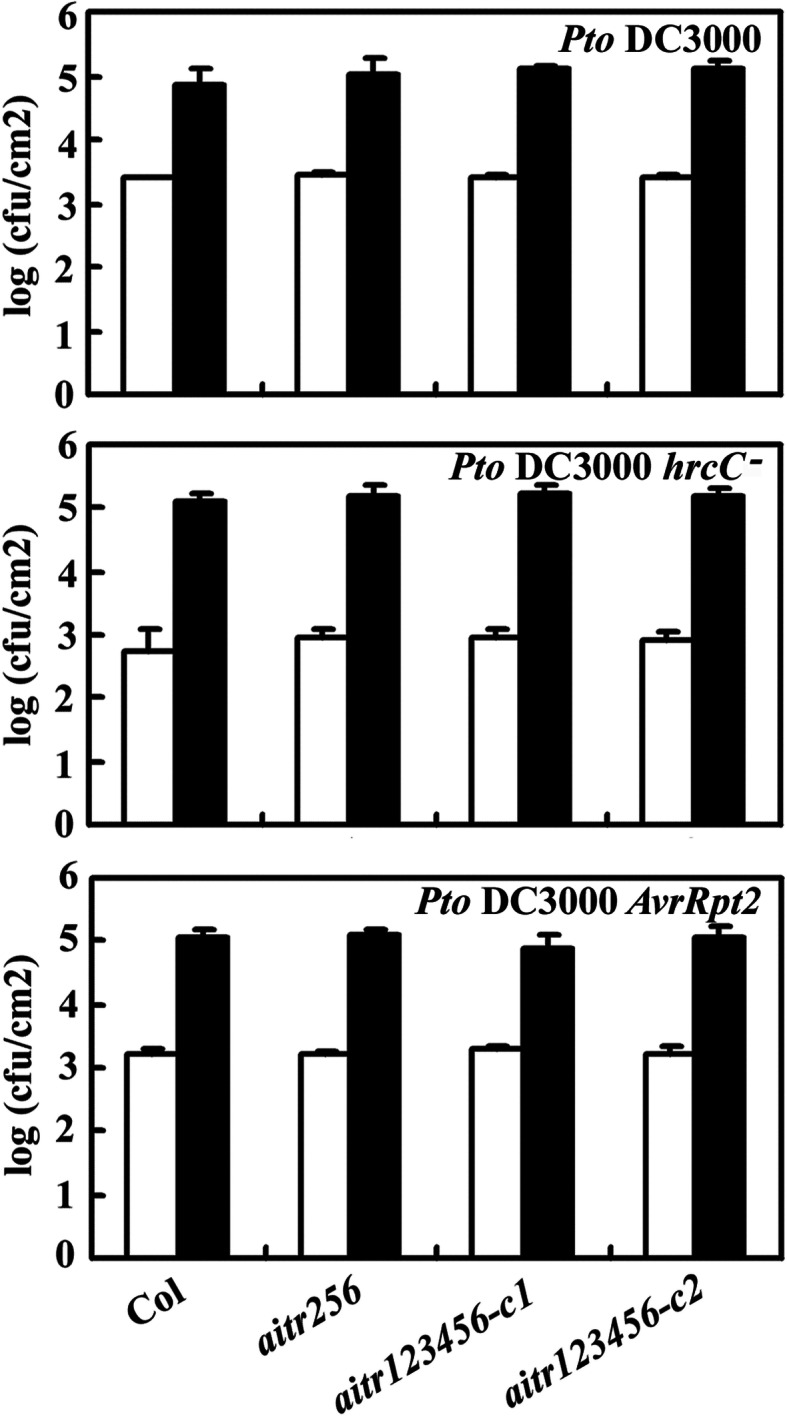
Response of the *aitr* mutants to pathogen infection. Seeds of the Col wild type and the *aitr256* triple and *aitr123456* sextuple mutants were germinated directly and grown in soil pots in a growth chamber at short-day condition. Plants about four-week-old were infiltrated with *Pto* DC3000, *Pto* DC3000 *hrcC*^−^ and *Pto* DC3000 *AvrRpt2* respectively. Samples were collected at day 0 and day 3, and bacterial growth was examined. The experiments were repeated twice, and similar results were obtained. Data represent the mean ± SD of 4 samples

The Col wild type plants and the *aitr* mutants were challenged with the virulent bacterial pathogen *Pseudomonas syringae* pv *tomato* (*Pto*) DC3000, *Pto* DC3000 *hrcC*^−^ and *Pto* DC3000 *AvrRpt2*, respectively, and growth of the bacterial was examined. As shown in Fig. 8, all the *aitr* mutants showed a response similar to that in the Col wild type plants.

## Discussion

Genome editing by CRISPR/Cas9 is able to generate transgene-free mutants in different plant species, and has been successfully used to improve important agronomic traits in several different crops [17, 20–22, 56]. However, identification of suitable candidate genes is a challenge for using CRISPR/Cas9 genome editing techniques in plant breeding. We provide evidence in this study that *AITR* genes maybe targeted for genome editing to improve plant tolerance to abiotic stresses without compromising their agronomic performance.

Environmental stresses, especially abiotic stresses such drought and salt are a global problem causing yield lost in plants including the most important crops [5, 6]. As a key stress hormone, ABA regulates plant tolerance to abiotic stresses [25–30]. As a result, changes the expression levels of the core regulator genes in ABA signaling usually affected plant responses to abiotic stresses. As examples, manipulation of the expression of the *PYL* receptor genes, the *SnRK2* protein kinase genes, and/or the *ABF*/*AREB* transcription factor genes affected drought tolerance in Arabidopsis [45, 46, 48]. However, enhanced abiotic tolerance are commonly observed in plants overexpressing these core regulator genes, whereas loss-of-function mutation usually led to decreased abiotic stress tolerance [45, 46, 48]. Therefore it is unlikely that these core regulator genes can be targeted by CRISPR/Cas9 for genome editing to enhance plant tolerance to abiotic stresses.

**Fig. 9:**
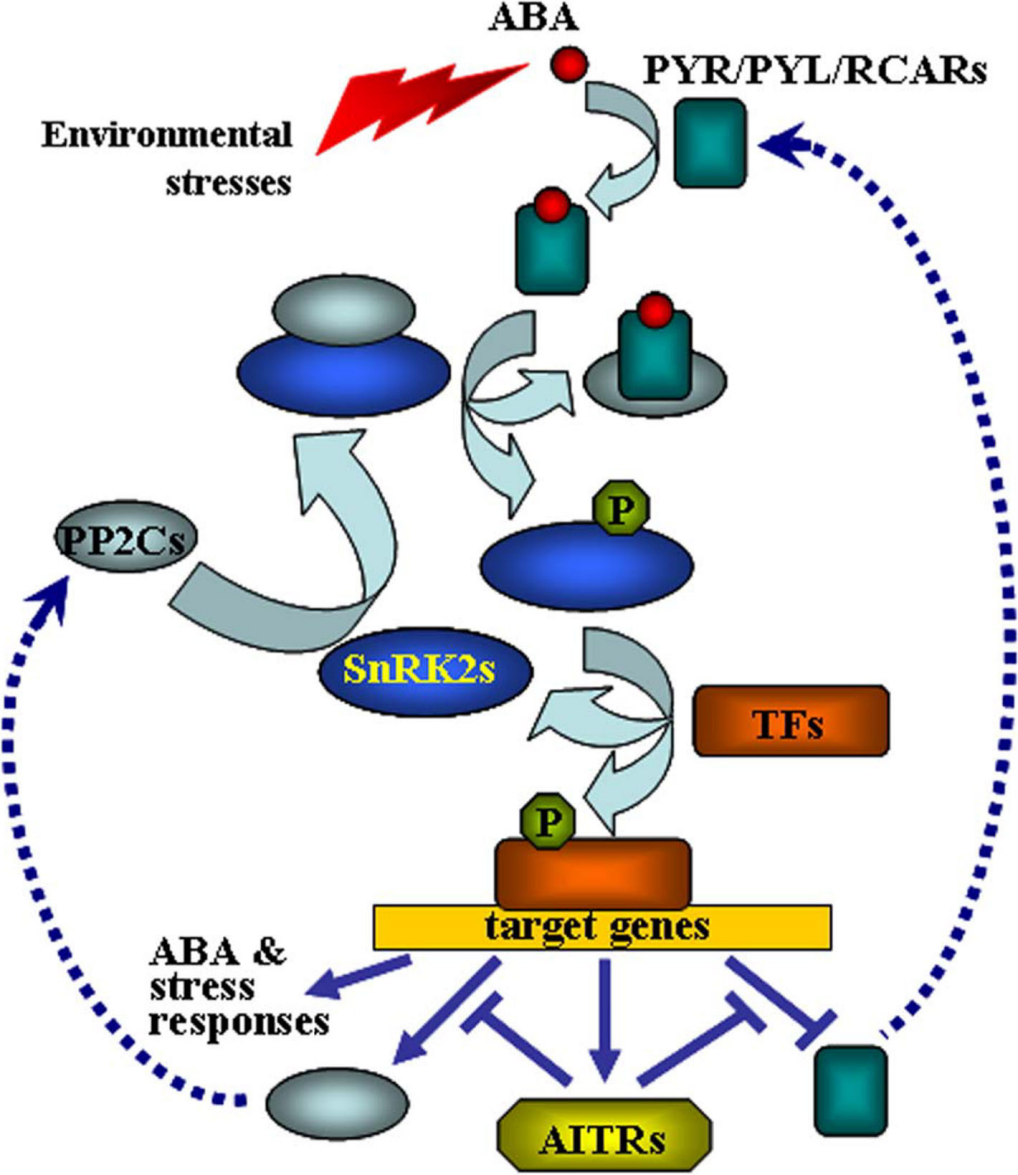
A model of action of AITRs in ABA and abiotic stress response. In the absent of ABA, PP2C phosphatases bind and deactivate SnRK2 kinases. Environmental stimuli and developmental cues promote ABA biosynthesis. ABA binds to PYR/PYL/RCAR receptors and suppresses the activity of PP2Cs. SnRK2s thus become activated, and they, in turn, phosphorylate down-stream transcription factors (TFs), resulting in the activation or repression of their target genes, including *PP2Cs*, *PYR/PYL/RCARs* and *AITRs*. Activation of *PP2Cs* and repression of *PYR/PYL/RCARs* represent negative feedback regulatory loops. AITRs regulate the expression of *PP2Cs* and *PYR/PYL/RCARs*, therefore function as feed back regulators in ABA signaling and abiotic stress responses

We have previously found that AITRs, a family of novel transcription factors function as feedback regulators in ABA signaling and plant response to abiotic stresses (Fig. 9), and loss-of-function of *AITRs* is able to enhance abiotic tolerance in Arabidopsis [30]. By using CRISPR/Cas9 to edit *AITR3* and *AITR4* in T-DNA insertation mutants *aitr256* and *aitr1256*, respectively, we generated *aitr23456* quintuple and *aitr123456* sextuple mutants (Fig. 1). We found that, in both seed germination and green cotyledon assays, all the *aitr* higher order mutants showed hyposensitivity to ABA (Figs. 2 and 3), and enhanced tolerance to drought and salt treatments (Figs. 5 and 6). However, when compared with the *aitr256* triple mutant, little if any enhanced ABA and abiotic stress tolerance were observed in the high order mutants including the *aitr123456* sextuple mutants, in which all the 6 *AITR* genes have been knocked out. Consistent with these observation, ABA response of the PYL receptor genes *PYL4*, *PYL5* and *PYL6*, the PP2C phosphatase gene *HAI1* and ABF/AREB/ABI5 transcription factor gene *ABF3*, and the SnRK2 kinase genes *SnRK2.2*, *SnRK2.3* and *SnRK2.6* in response to ABA remained largely similar in the *aitr123456* sextuple mutants and the *aitr256* triple and the *aitr1256* quadruple mutants (Fig. 4). This is likely because the basal expression levels of *AITRs* were very low [30], loss-of-function of *AITR2*, *AITR5* and *AITR6* may have already reached the expression level threshold where highest degree of redundancy functions of *AITRs* can be achieved. Nevertheless, these results suggest that *AITRs* could be good candidate genes for CRISPR/Cas9 genome editing to improve plant abiotic stress tolerance trait.

In order to survive under stress conditions, plants usually make trade-off between growth and development and stress tolerance, as a result, enhanced tolerance usually accompanied by a fitness penalty [49, 50, 57]. Therefore it is a challenge to improve plant stress tolerance without fitness costs. So far a few approaches have been used to overcome this problem. As examples, by using a pathogen-inducible promoter to drive the expression of *IPA1*(*Ideal Plant Architecture 1*), a transcription factor gene involved in the regulation of yield and immunity response in rice [58], enhanced disease resistance and increased yield were obtained in the transgenic rice plants [57]. By using a uORF (upstream open reading frame)-containing immune-inducible promoter to drive the expression of key immune regulator genes, improved disease resistance with no fitness costs was observed in Arabidopsis and rice [50]. However, these strategies are still relied on the generation of transgenic plants.

Recently, It has been reported MYC2-TARGETED BHLH1 (MTB1), MTB2 and MTB3, three JA (jasmonate)-inducible bHLH transcription factors functioned as negative regulators of JA mediated plant biotic stress response [59]. The *mtb* mutants generated by CRISPR/Cas9 genome editing showed enhance resistance to insect attack, but overall growth and development of the mutants was not affected [59]. To our knowledge, *MTBs* may be the first gene family identified so far that can be targeted by CRISPR/Cas9 to improve biotic stress tolerance in plants without fitness costs. Our results showed that AITRs functioned as negative regulators of ABA mediated plant abiotic stress response (Figs. 5 and 6). Knockout of all the 6 *AITR* genes did not affect plant growth and development (Fig. 7), and plant response to pathogen infection was also not affected in the *aitr123456* sextuple mutants (Fig. 8). These results suggest that *AITRs* may represent the first gene family identified so far that can be targeted by CRISPR/Cas9 to improve abiotic stress tolerance in plants without fitness costs.

Considering that AITRs are only presented in angiosperms including all the crop species, and AITRs examined in other plants such as soybean and tomato shared similar features with the Arabidopsis AITRs [30], it is likely that *AITRs* can be targeted for genome editing to improve abitic stress tolerance in crops without worrying about fitness costs. Considering that both ABA and JA signaling pathways are conserved in plants, it may be interesting to examine if simultaneously knock out of *AITRs* and *MTBs* may enhance biotic and abiotic stresses in crops. Considering that current crops varieties grown in different areas may have different desired agronomic traits such as high yield and good quality, but showed low tolerance to abiotic stresses, enome editing of *AITR* genes in these crops may enhance their abiotic stress tolerance without affecting other traits, therefore integrate the desired agronomic traits in crops. Most crops have multiple *AITR* genes [30], it may not easy to knockout all the *AITR* genes in a crop. However, our observation showed that there is no significant difference between the *aitr256* triple mutants and *aitr123456* quadruple mutants in response to abiotic stresses, suggest that knocking out a few *AITR* genes knockout may sufficient to improve abiotic stress tolerance in crops, which makes the editing of *AITRs* to improve abiotic stress tolerance in crops more practicable.

## Conclusions

In summary, we found that knock-out of a few *AITR* genes in Arabidopsis are sufficient to enhance abiotic stress tolerance, and knock-out the entitle family of *AITR* genes do not have fitness costs. Because AITRs family is conserved in crops with multiple encoding genes, *AITRs* may be good candidate genes for molecular breeding to improve abiotic stress tolerance in plants. This discovery is likely to usher a new wave of manipulation of plant abiotic stress tolerance in agricultural settings via CRISPR/Cas9 genome editing based molecular breeding.

## Methods

### Plant materials and growth conditions

The Col ecotype Arabidopsis stored in our laboratory was used as wild type, and the mutants were all in the Col ecotype background. The *aitr1* single and the *aitr2 aitr5 aitr6* (*aitr256*) triple mutants stored in our laboratory were as described previously [30]. The *aitr1 aitr2 aitr5 aitr6* (*aitr1256*) quadruple mutant was generated by crossing the *aitr1* single mutant and *aitr2 aitr5 aitr6* triple mutant, and genotyping the F2 progeny.

Arabidopsis seedlings used for ABA or abiotic treatments and RNA isolation were germinated on ½ MS plates. Arabidopsis plants used for genome editing, phenotypic observation and pathogen infection were germinated directly and grown in soil pots. All the plants were grown in a growth room with growth conditions described previously [30, 60], except that Arabidopsis plants used for pathogen infection were grown in a growth chamber at short-day conditions. Since the plants were grown by ourselves, we have all the right to collect the plants materials.

### Construct

CRISPR/Cas9 genome editing construct for *AITR3* and *AITR4* editing was generated using the *pHEE401E* vector. Appropriate target sites on the genome sequences of the single exon of *AITR3* and *AITR4* were identify on CRISPRscan (www.crisprscan.org), and then evaluated on Cas-OFFinder (www.rgenome.net/cas-offinder/). The target sequences used for *AITR3* and *AITR4* editing were 5’-GGGTAAACCGGGCCTACCGG(AGG)-3’, and 5’- TGGTTAACGAGGCTTACCGG(AGG)-3’, respectively. The CRISPR/Cas9 construct was generated by following a procedure described previously [61]. The primers used to insert the target sequences into the *pHEE401E* vector were, *AITR3*-*DT1*-*BsF*, 5’-ATATATGGTCTCGATTGGGTAAACCGGGCCTACCGGGTT-3’, *AITR3*-*DTI*-*F0*, 5’-TGGGTAAACCGGGCCTACCGGGTTTTAGAGCTAGAAATAGC-3’, *AITR4*-*DT2*-*R0*, 5-AACCCGGTAAGCCTCGTTAACCCAATCTCTTAGTCGACTCTAC-3’, and *AITR4*-*DT2*-*BsR*, 5’-ATTATTGGTCTCGAAACCCGGTAAGCCTCGTTAACCC-3’. The *U626*-*IDF* and *U629*-*IDR* primers used for clone PCR and sequencing of the sgRNA expression cassette in the CRISPR/Cas9 construct were described previously [62].

### Plant transformation, transgenic plant identification and Cas9-free mutant isolation

About 5-week-old *aitr256* triple and *aitr1256* quadruple mutant plants, when several mature flowers were produced on the main inflorescence stems, were used for transformation. Plants were transformed with the CRISPR/Cas9 construct generated by using the floral dip method [63]. To select transgenic plants, collected T1 seeds from the transformed plants were plated on 1/2 MS plates conaining 30 µg/ml hygromycin and 50 µg/ml carbenicillin. To examine gene editing status in the transgenic plants, genome sequences of *AITR3* and *AITR4* in T1 plants were amplified and sequenced. To select Cas9-free homozygous mutants, T2 progeny of confirmed gene edited T1 plants were germinated directly in soil pots, and gene editing status and absence of Cas9 were examined. Two confirmed independent homozygous Cas9-free mutants were used for the experiments.

### DNA isolation and PCR

To examine genome editing status of *AITR3* and *AITR4*, leaves of the T1 transgenic plants or T2 progeny of individual gene edited T1 plants were collected, and used for DNA isolation. Isolated DNA was used for PCR amplification of the genome sequence of *AITR3* and *AITR4*, respectively. PCR products was isolated and sent for sequencing, and sequencing results was aligned with the wild type sequences of *AITR3* and *AITR4*, respectively. To isolate Cas9-free mutants, leaves of the T2 progeny of individual gene edited T1 plants were collected, DNA was isolated and used to amply *Cas9* by PCR. The primers used for amplification of *AITR3* were, *AITR3*-*MF*, 5’-AATGGAGATAAAGCTGGTGAGT-3’ and *AITR3-R*, 5’- TCACATGCCAAGCCTTAGAG-3’. For *AITR4* were, *AITR4*-*MF*, 5’- TGGAGTCCGTTAACAGTGG-3’ and *AITR4*-*R*, 5’-TCAAAGCCAAAGAGT-3’. The *Cas9*-*F* and *Cas9*-*R* primers used for amplification of *Cas9* were described previously [62].

### RNA isolation and quantitative RT-PCR (qRT-PCR)

Twelve-day-old seedlings of the Col wild type, the *aitr256*, *aitr1256* and *aitr123456* mutants were used for ABA treatment as described previously [30]. Total RNA was isolated by using an EasyPure Plant RNA Kit (Transgene), and 1 µg RNA was subjected to cDNA synthesis by using an EazyScript First-Strand DNA Synthesis Super Mix Kit (TransGen Biotech).

The reponse of ABA signaling component genes in response to ABA treatment were examined by using qRT-PCR, and calculated as described previously [30]. All the primers used were as repotted previously [30, 64–67].

### ABA sensitivity analysis

Seed germination and cotyledon greening assays of ABA sensitivity were performed as described by previously [68]. Briefly, seeds of the Col wild type, the *aitr256* triple, *aitr1256* quadruple, *aitr23456* quintuple and *aitr123456* sextuple mutants were sterilized and plated on 1/2 MS plates with or without indicated concentrations of ABA. After kept in darkness at 4 °C for 2 days, the plates were transferred to a growth room.

For seed germination assays, seed germinated was examined and counted under a dissection microscopy daily after the transfer, and germination rate was calculated. For cotyledon greening assays, pictures of the seedlings were taken 17 days after the transfer, green seedlings were counted, and percentage of green seedlings was calculated.

### Drought tolerance analysis

Drought tolerance was assayed as previously described [30] with modifications. In brief, the seeds of the Col wild type and the *aitr256* triple, *aitr1256* quadruple, *aitr23456* quintuple and *aitr123456* sextuple mutants were germinated directly and grown in soil pots in a growth room for 30 days with normal watering. Pictures were taken and the plants were subjected to drought treated for 12 days by withholding watering. Pictures were taken and watering was resumed after drought treatment. Pictures were taken again 2 day after watering was resumed.

### Salt tolerance analysis

Salt tolerance of the *aitr* mutants was assayed as previously described [30] with modifications. In brief, seeds of the Col wild type and the *aitr256* triple, *aitr1256* quadruple, *aitr23456* quintuple and *aitr123456* sextuple mutants were germinated directly and grown in soil pots in a growth room for 30 days with sufficient watering. Pictures were taken and the plants were then watered every other day with 150 mM NaCl for 10 times. Pictures were taken after salt treatment.

### Pathogen infection assays

For pathogen infection assays, seeds of the Col wild type and the *aitr123456* sextuple mutants were germinated directly and grown in soil pots in a growth chamber at short-day condition. Plants ~ 4-week-old were infiltrated and bacterial growth was assayed as described previously [69]. The pathogens used for infiltration and their corresponding concentrations were *Pseudomonas syringae* pv *tomato* (*Pto*) DC3000, *Pto* DC3000 *hrcC*^−^ and *Pto* DC3000 *AvrRpt2* at a dose of OD_600_ = 0.0002, 0.001 and 0.001, respectively.

### Morphological assays

For plant growth and development assays, seeds of the Col wild type and the *aitr256* triple, *aitr1256* quadruple, *aitr23456* quintuple and *aitr123456* sextuple mutants were germinated directly and grown in soil pots in a growth room. Morphology of the plants at different growth stages was examined. Pictures for the plants and inflorescences at indicated growth stages were taken by using a digital camera, and pictures for seeds were taken under a dissection microscopy equipped with a digital camera.

## Data Availability

All data generated or analysed during this study are included in this published article.

## Abbreviations

ABA: Abscisic acid
AITRs: ABA-induced transcription repressors
bZIP: Basic region leucine zipper
CRISPR/Cas9: Clustered regularly interspaced short palindromic repeats/CRISPR -associated protein 9
IPA1: Ideal Plant Architecture 1
JA: Jasmonate
KEG: KEEP ON GOING
MTB1: MYC2-TARGETED BHLH1
PP2Cs: PROTEIN PHOSPHATASE 2C
PYL: PYR1-like
PYR1: Pyrabactin resistance 1
RCAR: Regulatory component of ABA receptor
SnRKs: NONFERMENTING 1 (SNF1)-RELATED PROTEIN KINASES
uORF: Upstream open reading frame
